# Crystal structure of SEL1L: Insight into the roles of SLR motifs in ERAD pathway

**DOI:** 10.1038/srep20261

**Published:** 2016-02-09

**Authors:** Hanbin Jeong, Hyo Jung Sim, Eun Kyung Song, Hakbong Lee, Sung Chul Ha, Youngsoo Jun, Tae Joo Park, Changwook Lee

**Affiliations:** 1 Department of Biological Sciences, School of Life Sciences, Ulsan National Institute of Science and Technology, 50 UNIST-gil, Ulsan 44919, Republic of Korea; 2 Pohang Accelerator Laboratory, Pohang University of Science and Technology, Pohang, Kyungbuk 37673, Korea; 3 School of Life Sciences, Gwangju Institute of Science and Technology, Gwangju 61005, Korea

## Abstract

Terminally misfolded proteins are selectively recognized and cleared by the endoplasmic reticulum-associated degradation (ERAD) pathway. SEL1L, a component of the ERAD machinery, plays an important role in selecting and transporting ERAD substrates for degradation. We have determined the crystal structure of the mouse SEL1L central domain comprising five Sel1-Like Repeats (SLR motifs 5 to 9; hereafter called SEL1L^cent^). Strikingly, SEL1L^cent^ forms a homodimer with two-fold symmetry in a head-to-tail manner. Particularly, the SLR motif 9 plays an important role in dimer formation by adopting a domain-swapped structure and providing an extensive dimeric interface. We identified that the full-length SEL1L forms a self-oligomer through the SEL1L^cent^ domain in mammalian cells. Furthermore, we discovered that the SLR-C, comprising SLR motifs 10 and 11, of SEL1L directly interacts with the N-terminus luminal loops of HRD1. Therefore, we propose that certain SLR motifs of SEL1L play a unique role in membrane bound ERAD machinery.

Protein quality control in the endoplasmic reticulum (ER) is essential for maintenance of cellular homeostasis in eukaryotes and is implicated in many severe diseases^1^. Terminally misfolded proteins in the lumen or membrane of the ER are retrotranslocated into the cytosol, polyubiquitinated, and degraded by the proteasome. The process is called ER-associated protein degradation (ERAD) and is conserved in all eukaryotes^2^,^3^,^4^. Accumulating studies have identified key components for ERAD, including HRD1, SEL1L (Hrd3p), Derlin-1, -2, -3 (Der1p), HERP-1, -2 (Usa1p), OS9 (Yos9), XTP-B, and Grp94, all of which are involved in the recognition and translocation of the ERAD substrates in yeast and metazoans^5^,^6^,^7^,^8^,^9^. The components are differentially localized from the lumen and membrane of the ER to the cytosol, and have different functions in the ERAD process. Yeast ERAD components, which have been extensively characterized through genetic and biochemical studies, are comparable with mammalian ERAD components, sharing similar molecular functions and structural composition.

The HRD1 E3 ubiquitin ligase, which is embedded in the ER membrane, is involved in translocating ERAD substrates across the ER membrane and catalyzing substrate ubiquitination via its cytosolic RING finger domain^10^. SEL1L, the mammalian homolog of Hrd3p, associates with HRD1, mediates HRD1 interactions with the ER luminal lectin OS9, and recognizes substrates to be degraded^6^,^11^,^12^,^13^,^14^,^15^. In particular, SEL1L is crucial for translocation of Class I major histocompatibility complex (MHC) heavy chains (HCs)^14^,^15^. Recent research based on the inducible *Sel1l* knockout mouse model highlights the physiological functions of SEL1L^16^,^17^. SEL1L is required for ER homeostasis, which is essential for protein translation, pancreatic function, and cellular and organismal survival. However, despite the functional importance of SEL1L, the molecular structure of SEL1L has not been solved. Previous biochemical studies reveal that SEL1L is a type I transmembrane protein and has a large luminal domain comprising sets of repeated Sel1-like (SLR) motifs^18^. The SLR motif is a structural motif that closely resembles the tetratricopeptide-repeat (TPR) motif, which is a protein-protein interaction module^19^. Although there is evidence that the luminal domain of SEL1L is involved in substrate recognition or in forming complexes with chaperones^12^, it is not known how the unique structure of the repeated SLR motifs contributes to the molecular function of the HRD1-SEL1L E3 ligase complex and affects ERAD at the molecular level. Furthermore, while repeated SLR motifs are commonly found in tandem arrays, the SLR motifs in SEL1L are, according to the primary structure prediction of SEL1L, interspersed among other sequences in the luminal domain and form three SLR domain clusters. Therefore, the way in which these unique structural features of SEL1L are related to its critical function in ERAD remains to be elucidated.

To clearly understand the biochemical role of the SLR domains of SEL1L in ERAD, we determined the crystal structure of the central SLR domain of SEL1L. We found that the central domain of SEL1L, comprising SLR motifs 5 through 9 (SEL1L^cent^), forms a tight dimer with two-fold symmetry due to domain swapping of the SLR motif 9. We also found that SLR-C, consisting of SLR motifs 10 and 11, directly interacts with the N-terminus luminal loop of HRD1. Based on these observations, we propose a model wherein the SLR domains of SEL1L contribute to the formation of stable oligomers of the ERAD translocation machinery, which is indispensable for ERAD.

## Results

### Structure Determination of SEL1L^cent^

**Figure 1:**
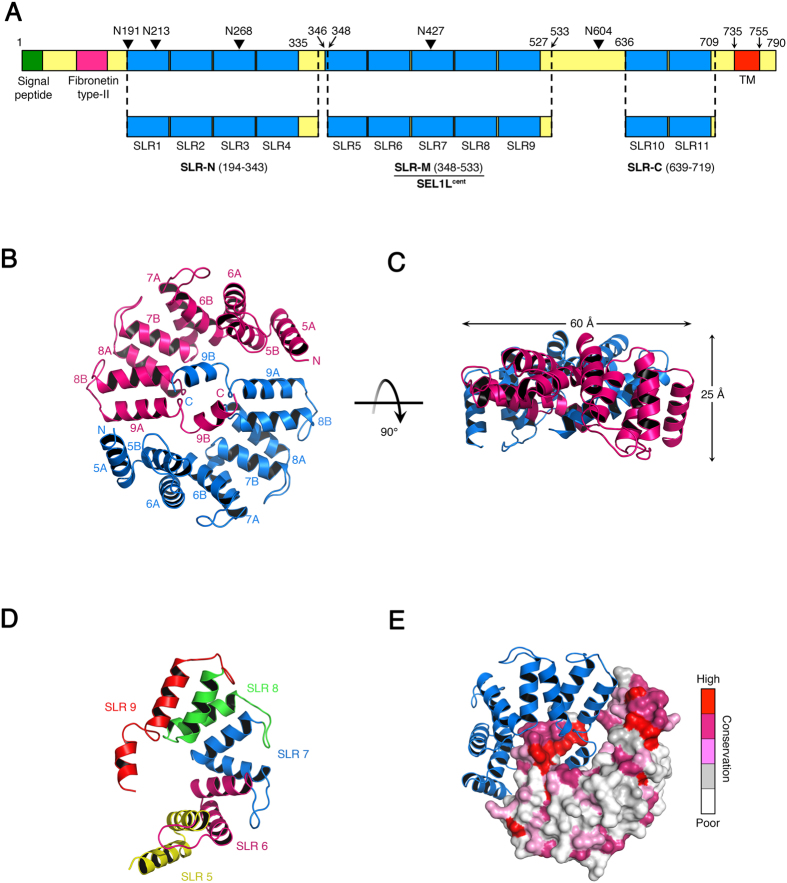
Crystal Structure of SEL1L^cent^. (**A**) The diagram shows the domain structure of *Mus musculus* SEL1L, as defined by proteolytic mapping and sequence/structure analysis. The 11 SLR motifs were divided into three groups (SLR-N, SLR-M, and SLR-C) due to the presence of linker sequences that are not predicted SLR motifs. Putative N-glycosylation sites are indicated by black triangles. We determined the crystal structure of the SLR-M, residues 348–533. (**B**) Ribbon diagram of the biological unit of the SEL1L^cent^, viewed along the two-fold NCS axis. The crystal structure was determined by SAD phasing using selenium as the anomalous scatterer and refined to 2.6 Å resolution (Table 1). (**C**) SEL1L^cent^ ribbon diagram rotated 90° around a horizontal axis relative to (**B**). (**D**) One protomer of the SEL1L^cent^ dimer. This view is rotated about 90° anticlockwise from the bottom copy in (**B**), along the two-fold NCS axis. Starting from the N-terminus, SEL1L^cent^ has five SLR motifs comprising ten α helices. Each SLR motif (from 5 to 9) is indicated in a different color. (**E**) Evolutionary conservation of surface residues in SEL1L^cent^, calculated using ConSurf 22, from a structure-based alignment of 135 SEL1L sequences. The surface is colored from red (high) to white (poor) according to the degree of conservation in the SEL1L phylogenetic orthologs. The ribbon diagram of the counterpart protomer is drawn to show the orientation of the SEL1L^cent^ dimer.

The *Mus musculus* SEL1L protein contains 790 amino acids and has 17% sequence identity to its yeast homolog, Hrd3p. Mouse SEL1L contains a fibronectin type II domain at the N-terminus, followed by 11 SLR motifs and a single transmembrane domain at the C-terminus (Fig. 1A)^18^. The 11 SLR motifs are located in the ER lumen and account for more than two thirds of the mass of full-length SEL1L. The SLR motifs can be grouped into three regions due to the presence of linker sequences among the groups of SLR motifs: SLR-N (SLR motifs 1 to 4), SLR-M (SLR motifs 5 to 9), and SLR-C (SLR motifs 10 to 11) (Fig. 1A). Sequence alignment of the SLR motifs revealed that there is a short linker sequence (residues 336–345) between SLR-N and SLR-M and a long linker sequence (residues 528–635) between SLR-M and SLR-C (Fig. 1A). We first tried to prepare the full-length mouse SEL1L protein, excluding the transmembrane domain at the C-terminus (residues 735–755), by expression in bacteria. However, the full-length SEL1L protein aggregated in solution and produced no soluble protein. To identify a soluble form of SEL1L, we generated serial truncation constructs of SEL1L based on the predicted SLR motifs and highly conserved regions across several different species. Both SLR-N (residues 194–343) and SLR-C (residues 639–719) alone could be solubilized with an MBP tag at the N-terminus, but appeared to be polydisperse when analyzed by size-exclusion chromatography. However, the central region of SEL1L, comprising residues 337–554, was soluble and homogenous in size, as determined by size-exclusion chromatography. To define compact domain boundaries for the central region of SEL1L, we digested the protein with trypsin and analyzed the proteolysis products by SDS-PAGE and N-terminal sequencing. The results of this preliminary biochemical analysis suggested that SEL1L residues 348–533 (SEL1L^cent^) would be amenable to structural analysis (Fig. 1A).

Crystals of SEL1L^cent^ grew in space group P2_1_ with four copies of SEL1L^cent^ (a total of 82 kDa) in the asymmetric unit. The structure was determined by the single-wavelength anomalous diffraction (SAD) method using selenium as the anomalous scatterer (Table 1 and Methods). The assignment of residues during model building was aided by the selenium atom positions, and the structure was refined with native data to 2.6 Å resolution with *R*_work_/*R*_free_ values of 20.7/27.7%. Statistics for data collection and refinement are presented in Table 1.

### Overall Structure of SEL1L^cent^

The mouse SEL1L^cent^ crystallized as a homodimer, and there were two homodimers in the crystal asymmetric unit (Fig. 1B,C, Supplementary Fig. 1). The two SEL1L^cent^ molecules dimerize in a head-to-tail manner through a two-fold symmetry interface resulting in a cosmos-like shaped structure (Fig. 1B). The resulting structure resembles the yin-yang symbol with overall dimensions of 60 × 60 × 25 Å, where a SEL1L^cent^ monomer corresponds to half the symbol. The dimer formation buries a surface area of 1670 Å^2^ for each monomer, and no significant differences between the protomers were displayed (final root mean square deviation (RMSD) of 0.6 Å for all Cα atoms). Each protomer is composed of ten α-helices, which form the five SLRs, resulting in an elongated curved structure, confirming the primary structure prediction (Fig. 1D).

The α-helices subdivide the structure into five pairs (A and B) as shown in a number of TPRs^19^ and SLRs^20^,^21^. Helices A and B are 14 and 13 residues long, respectively, and the two helices are connected by a short turn and loop (Fig. 1D). In addition, a longer loop, consisting of approximately eight amino acids, is inserted between helix B of one SLR and helix A of the next SLR. This arrangement is a unique feature for SLRs among the major classes of repeats containing an α-solenoid. Starting from its N-terminus, the α-solenoid of SEL1L extends across a semi-circle in a right-handed superhelix fashion along the rotation axis of the yin-yang circle. However, the last helix, 9B, at the C-terminus adopts a different conformation, lying parallel to the long axis of helix 9A instead of forming an antiparallel SLR. This unique conformation of helix 9B most likely contributes to formation of the dimer structure of SEL1L^cent^, as detailed below. With the exception of the last SLR, the four α-helix pairs possess similar conformations, with RMSD values of 0.7 Å for all Cα atoms. Although the sequence similarity for the pairwise alignments varies between 25% and 35%, all the residues present in the SLR motifs are conserved among the five pairs. The SLR domain of SLR-M ends at residue 524, and C-terminal amino acids 525–533 of the protein are not visible in the electron density map, suggesting that this region is highly flexible.

Since no information regarding dimer formation by SEL1L through its SLR motifs is available, we tested whether the SEL1L^cent^ dimer shown in our crystal structure is a biological unit. First, we cross-linked SEL1L^cent^ or a longer construct of SEL1L (SEL1L^long^, residues 337–554) using various concentrations of glutaraldehyde (GA) or dimethyl suberimidate (DMS) and analyzed the products by SDS-PAGE. We detected bands at the mass of a dimer for both SEL1L^cent^ and SEL1L^long^ when cross-linked with low concentrations of GA (0.005%) or DMS (0.3 mM) (Supplementary Fig. 2A,B). Next, we conducted analytical ultracentrifugation of SEL1L^cent^. Consistent with the cross-linking data, analytical ultracentrifugation revealed that the molecular weight of SEL1L^cent^ corresponds to a dimer (Supplementary Fig. 2C). Taken together, these data indicate that some kind of dimer is formed in solution.

### Dimer Interface of SEL1L^cent^

**Figure 2:**
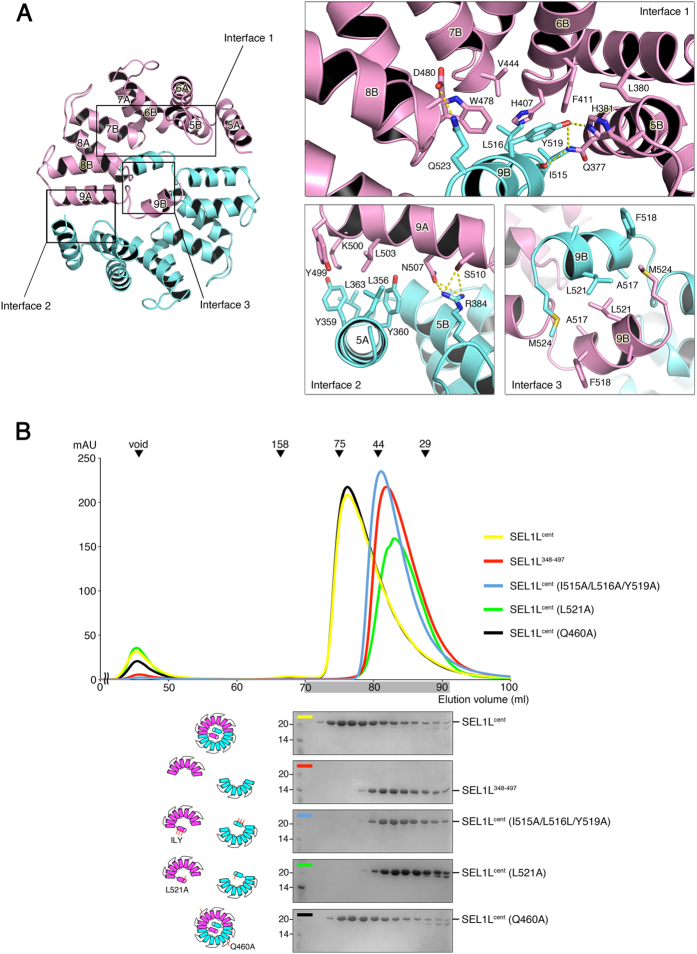
Dimer Interface of SEL1L^cent^. (**A**) The diagram on the left shows the SEL1L^cent^ dimer viewed along the two-fold symmetry axis. Three distinct contact regions are indicated with labeled boxes. The close-up view on the right shows the residues of SEL1L^cent^ that contribute to dimer formation via the three contact interfaces. Oxygen and nitrogen atoms are shown as red and blue, respectively. The yellow dotted lines indicate intermolecular hydrogen bonds between two protomers of SEL1L^cent^. (**B**) Size-exclusion chromatography (SEC) analysis of the wild-type and dimeric interface SEL1L^cent^ mutants to compare the oligomeric states of the proteins. The standard molecular masses for the SEC experiments (top) were obtained from the following proteins: aldolase, 158 kDa; cobalbumin, 75 kDa; ovalbumin, 44 kDa; and carbonic anhydrase, 29 kDa. Chromatography was performed on a Superdex 200 column with a buffer containing 25 mM Tris, 150 mM NaCl, and 5 mM DTT (pH 7.5). The elution fractions, indicated by the gray shading, were run on SDS-PAGE and are shown below the gel-filtration elution profile. The schematic diagrams representing the protein constructs used in the SEC are shown on the left of each SDS-PAGE profile.

In contrast to a previously described SLR motif containing proteins that exist as monomers in solution^20^,^21^, SEL1L^cent^ forms an intimate two-fold homotypic dimer interface (Figs 1B and 2A). The concave surface of each SEL1L domain comprising helix 5A to 9A encircles its dimer counterpart in an interlocking clasp-like arrangement. However, no interactions were seen between the two-fold-related protomers through the concave inner surfaces themselves. Rather, the unique structure of SLR motif 9, consisting of two parallel helices (9A and 9B), is located in the space generated by the concave surface and provides an extensive dimerization interface between the two-fold-related molecules (Fig. 2A). Helix 9B from one protomer inserts into the empty space surrounded by the concave region in the other monomer, forming a domain-swapped conformation.

Three major contact interfaces are involved in the interactions, and all interfaces are symmetrically related between the dimer subunits (Fig. 2A). Structure-based sequence alignment of 135 SEL1L phylogenetic sequences using a ConSurf server revealed that the surface residues in the dimer interfaces were highly conserved among the SEL1L orthologs (Fig. 1E)^22^. First, helix 9B of each SEL1L^cent^ subunit interacts with residues lining the inner groove from the SLR α-helices (5B, 6B, 7B, and 8B) from its counterpart. In this interface, Leu 516 and Tyr 519 on helix 9B are located in the center, making hydrophobic interactions with Trp 478 on helix 8B, Val 444 on helix 7B, Phe 411 on helix 6B, and Leu 380 on helix 5B from the SEL1L^cent^ counterpart (Fig. 2A, *Interface 1 detail*). In addition to hydrophobic interactions, the side chain hydroxyl group of Tyr 519 and the main-chain oxygen of Ile 515 form H-bonds to the side chain of the conserved Gln 377 and His 381 on helix 5B of the two-fold-related protomer. The side chain of Gln 523 forms an H-bond to the side chain of Asp 480 on the two-fold-related protomer (Fig. 2A, *Interface 1 detail*). Second, the residues from helix 9A interact with the residues from helix 5A of its counterpart in a head-to-tail orientation. In this interface, the interacting residues on helix 9A, including Leu 503, Tyr 499, and the aliphatic side chain of Lys 500, form an extensive network of van der Waals contacts with the hydrophobic residues of the counterpart helix 5A, including Tyr 360, Leu 356, Tyr 359, and Leu 363. In addition to hydrophobic interactions, the side chains of Asn 507 and Ser 510 on helix 9A make H-bonds with highly conserved Arg 384 in the loop between helix 5B and 6A from the two-fold-related protomer (Fig. 2A, *Interface 2 detail*). Third, the helix 9B from each protomer is involved in the dimer interaction by forming a two-fold antiparallel symmetry. In particular, the side chains of hydrophobic residues, including Phe 518, Leu 521, and Met 524, are directed toward each other, where they make both inter- and intramolecular contacts (Fig. 2A, *Interface 3 detail*).

To further investigate the interactions observed in our crystal structure, we generated a C-terminal deletion mutant (SEL1L^348–497^) lacking SLR motif 9 (helix 9A and 9B) from SEL1L^cent^ for comparative analysis. The deletion mutant and the wild-type SEL1L^cent^ showed no difference in spectra by CD spectroscopy, indicating that the deletion of the SLR motif 9 did not affect the secondary structure of SEL1L^cent^ (Supplementary Fig. 3). However, the mutant behaved as a monomer in size-exclusion chromatography and analytical ultracentrifugation experiments (Fig. 2B, Supplementary Fig. 2C). Additionally, to further validate the key residues involved in dimer formation, we generated a triple point mutant (*Interface 1*, I515A, L516A, and Y519A) of the hydrophobic residues that are involved in dimerization. The triple point mutant eluted at the monomer position upon size-exclusion chromatography, while the negative control point mutant (Q460A) eluted at the same position as the wild-type. Notably, a single-residue mutation (L521A in interface 3) abolished the dimerization of SEL1L^cent^ (Fig. 2B). Leu 521 is located in the dimerization center of the antiparallel 9B helices in the SEL1L^cent^ dimer.

Taken together, these structural and biochemical data demonstrate that SEL1L^cent^ exists as a dimer in solution and that SLR motif 9 in SEL1L^cent^ plays an important role in generating a two-fold dimerization interface.

### The Two Glycine Residues (G512 and G513) Create a Hinge for Domain Swapping of SLR Motif 9

**Figure 3:**
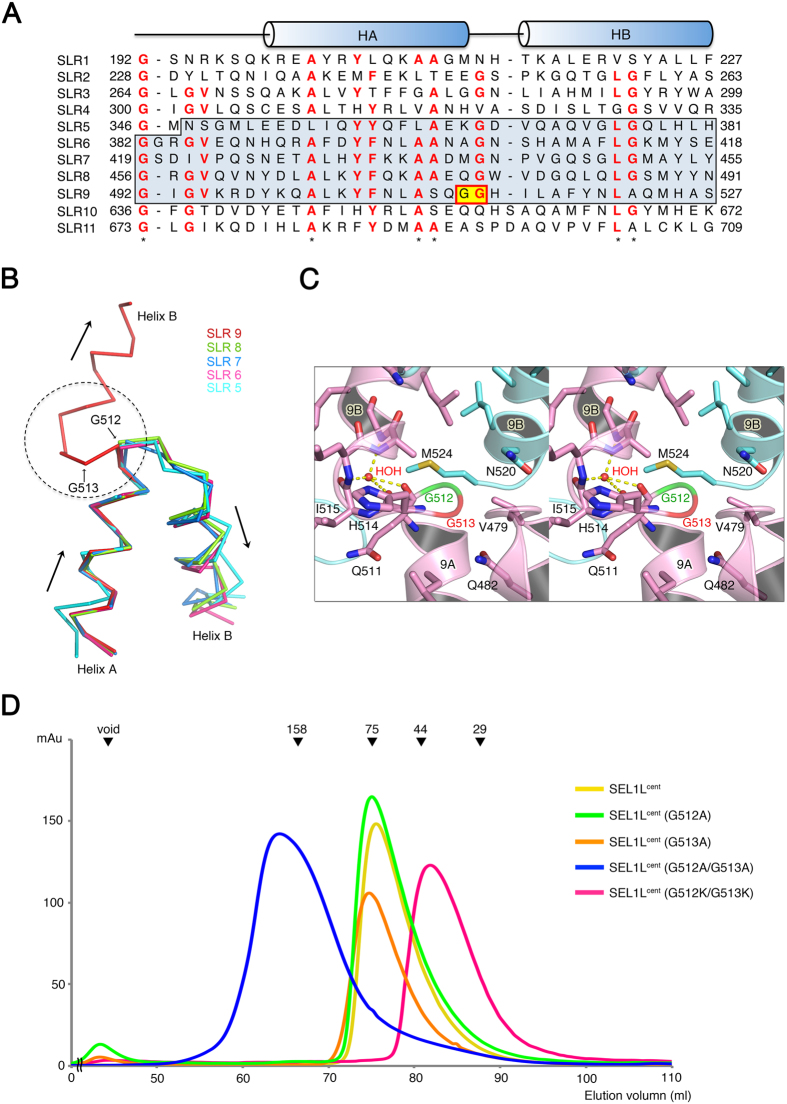
Domain Swapping for Dimerization of SEL1L^cent^. (**A**) Sequence alignment of the SLR motifs in SEL1L. The 11 SLR motifs were aligned based on the present crystal structure of SEL1L^cent^. The sequences of SEL1L^cent^ included in the crystal structure are highlighted by the blue box. The secondary structure elements are indicated above the sequences, with helices depicted as cylinders. Residues that are conserved in at least 7 out of 11 sequences are red. The GG sequence in SLR motif 9, which creates the hinge for domain swapping (see text), is shaded yellow. Stars below the sequences indicate the specific residues that commonly appear in SLRs. (**B**) Structure alignment of five SLR motifs in SEL1L^cent^ is shown to highlight the unusual geometry of SLR motif 9. Each SLR motif is shown in a different color. The arrow indicates the direction of the helical axes. In SLR motif 9, the axes for the two helices are almost parallel, while the other SLR motifs adopt an α-hairpin structure. (**C**) Stereo view shows that the Gly 512 and Gly 513 residues are surrounded by neighboring residues from helix 9B from the counterpart dimer. Oxygen and nitrogen atoms are colored red and blue, respectively. The Gly 512 and Gly 513 residues are colored green and red, respectively. (**D**) The following point mutations were generated to check the effect of the Gly 512 and Gly 513 residues in terms of generating the hinge of SLR motif 9: G512A, G513A, G512A/G513A, and G512K/G513K. Size-exclusion chromatography was conducted as described in Fig. 2B. The standard molecular masses are shown at the top as in Fig. 2B.

SLRs of mouse SEL1L were predicted using the TPRpred server^23^. Based on the prediction, full-length SEL1L contains a total of 11 SLR motifs, and our construct corresponds to SLR motifs 5 through 9. Although amino acid sequences from helix 9A and 9B correctly aligned with the regular SLR repeats and corresponded to SLR motif 9 (Fig. 3A), the structural arrangement of the two helices deviated from the common structure for the SLR motif. According to our crystal structure, the central axis of helix 9B is almost parallel to that of helix 9A (Fig. 3B). However, this unusual conformation of SLR motif 9 seems to be essential for dimer formation, as described earlier. For this structural geometry, two adjacent residues, Gly 512 and Gly 513, in SEL1L confer flexibility at this position by adopting main-chain dihedral angles that are disallowed for non-glycine residues. The phi and psi dihedrals are 100° and 20° for Gly 512, and 110° and −20° for Gly 513, respectively (Fig. 3C). Gly 513 is conserved among other SLR motifs in the SEL1L^cent^, but Gly 512 is present only in the SLR motif 9 of SEL1L^cent^ (Fig. 3A). Thus, the Gly-Gly residues generate an unusual sharp bend at the C-terminal SLR motif 9. The involvement of a glycine residue in forming a hinge for domain swapping has been reported previously^24^. The significance of Gly 513 is further highlighted by its absolute conservation among different species, including the budding yeast homolog Hrd3p.

To further investigate the importance of Gly 512 and Gly 513 in the unusual SLR motif geometry, we generated a point mutation (Gly to Ala), which restricts the flexibility. Although the Gly 512 and Gly 513 residues are closely surrounded by helix 9B from the counter protomer, there is enough space for the side chain of alanine, suggesting that no steric hindrance would be caused by the mutation (Fig. 3C). This means that the effect of the mutation is mainly to generate a more restricted geometry at the hinge region. G512A or G513A alone showed no differences from wild-type in terms of the size-exclusion chromatography elution profile (Fig. 3D), suggesting that the restriction for single glycine flexibility would not be enough to break the swapped structure of helix 9B. However, the double mutant (G512A/G513A) eluted over a broad range and much earlier than the wild-type, suggesting that mutation of the residues involved in the hinge linking helix 9A and 9B significantly affected the geometry of helix 9B in generating domain swapping, and eventually altered the overall oligomeric state of SEL1L^cent^ into a polydisperse pattern (Fig. 3D, Supplementary Fig. 6). When the residues were mutated to lysine (G512K/G513K), the mutant not only restricted the geometry of residues at the hinge but also generated steric hindrance during interaction with the counter protomer of SEL1L^cent^, thereby inhibiting self-association of SEL1L^cent^ completely. The G512K/G513K double mutant eluted at the monomer position in size-exclusion chromatography (Fig. 3D). A previous study shows that induction of steric hindrance by mutation destabilizes the dimerization interface of a different protein, ClC transporter^25^.

Collectively, these data suggest that the Gly 512 and Gly 513 at the connection between helix 9A and 9B play a crucial role in forming the domain-swapped conformation that enables dimer formation.

### SEL1L Forms Self-oligomers through SEL1L^cent^ domain *in vivo*

**Figure 4:**
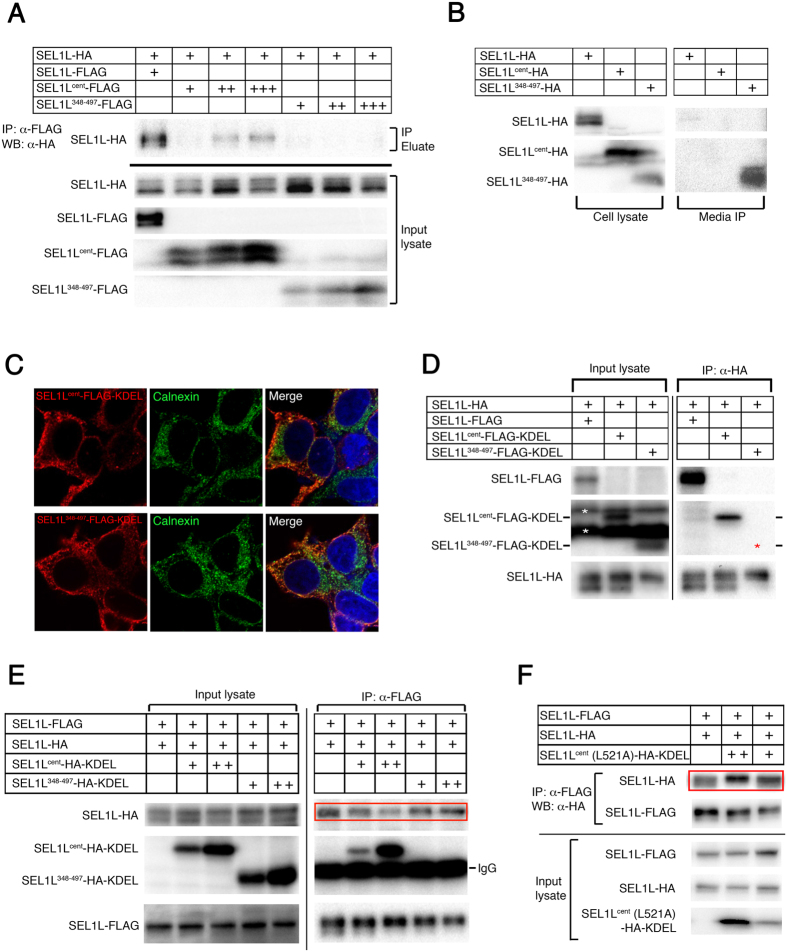
SEL1L forms self-oligomer mediated by the SEL1L^cent^ domain *in vivo.* (**A**) HEK293T cells were transfected with the indicated plasmid constructs and the lysates were immunoprecipitated with an anti-FLAG antibody followed by western blot analysis using an anti-HA antibody. The full-length SEL1L-FLAG was co-immunoprecipitated with the full-length SEL1L-HA. Also, SEL1L^cent^ was co-immunoprecipitated with the full-length SEL1L while the SLR motif 9 deletion failed to do so. (**B**) The HEK293T cells were transfected with the indicated plasmid constructs and the cell lysate and culture media were analyzed by western blot analysis and immunoprecipitation respectively. The SEL1L^348–497^ fragment was secreted to the culture media but the SEL1L^cent^ was retained in the ER. (**C**) SEL1L^cent^-FLAG-KDEL and SEL1L^348–497^-FLAG-KDEL localized to the ER. The nuclei were stained with DAPI in blue. The ER was visualized with the anti-calnexin antibody in green. The SEL1L fragments were stained in red. (**D**) HEK293T cells were transfected with the indicated plasmid constructs and the lysates were immunoprecipitated with an anti-HA antibody followed by Western blot analysis using an anti-FLAG antibody. The full-length SEL1L forms self-oligomers and the SEL1L^cent^-FLAG-KDEL was co-immunoprecipitated with full-length SEL1L-HA. The red asterisk indicates the expected signal for SEL1L^348–497^-FLAG-KDEL. SEL1L^348–497^-FLAG-KDEL did not co-immunoprecipitate with full-length SEL1L-HA. The white asterisks indicate non-specific bands. (**E**) SEL1L^cent^-HA-KDEL competitively inhibited self-oligomerization of full-length SEL1L. The indicated plasmid constructs were transfected and immunoprecipitation assay was performed using an anti-FLAG antibody followed by western blot analysis using an anti-HA antibody. The red rectangle indicates competitively inhibited SEL1L self-oligomer formation by the increasing doses of SEL1L^cent^-HA-KDEL. (**F**) L521A point mutant in SEL1L^cent^ did not inhibit the self-association of SEL1L.

Next, we examined if SEL1L also forms self-oligomers *in vivo* using HEK293T cells. We generated full-length SEL1L-HA and SEL1L-FLAG fusion constructs and co-transfected the constructs into HEK293T cells. A co-immunoprecipitation assay using an anti-FLAG antibody followed by Western blot analysis using an anti-HA antibody showed that full-length SEL1L forms self-oligomers *in vivo* (Fig. 4A). To further examine whether the SEL1L^cent^ domain is sufficient to physically interact with full-length SEL1L, we generated SEL1L^cent^ and SLR motif 9 deletion (SEL1L^348–497^) construct, which were fused to the C-terminus of SEL1L signal peptides. Co-immunoprecipitation analysis showed that the SEL1L^cent^ was sufficient to physically interact with the full-length SEL1L, while SEL1L^348–497^ failed to do so (Fig. 4A). Interestingly, however, the expression level of SEL1L^348–497^ was consistently lower than that of SEL1L^cent^ (Fig. 4A,B). Semi-quantitative RT-PCR revealed no significant difference in transcriptional levels of the two constructs (data not shown). We speculated that SEL1L^348–497^ could be secreted while the SEL1L^cent^ is retained in the ER by association with the endogenous ERAD complex. Indeed, immunoprecipitation followed by western blot analysis using the culture medium detected secreted SEL1L^348–497^ fragment, but not SEL1L^cent^ (Fig. 4B). We next examined if the reason why SEL1L^348–497^ failed to bind to the full-length SEL1L may be because of the lower level of SEL1L^348–497^ in the ER lumen compared to SEL1L^cent^ fragment. In order to retain two SEL1L fragments in the ER lumen, we added KDEL ER retention sequence to the C-terminus of both fragments. Indeed, the addition of KDEL peptide increased the level of SEL1L^348–497^ in the ER lumen (Fig. 4D,E) and the immunostaining analysis showed both constructs were well localized to the ER (Fig. 4C). We further analyzed whether SEL1L^cent^ may competitively inhibit the self-oligomerization of SEL1L *in vivo*. To this end, we co-transfected the differentially tagged full-length SEL1L (SEL1L-HA and SEL1L-FLAG) and increasing doses of SEL1L^cent^-KDEL, SEL1L^348–497^-KDEL or SEL1L^cent^ (L521A)-KDEL, respectively. Co-immunoprecipitation assay revealed that wild-type SEL1L^cent^-KDEL, indeed, competitively disrupted the self-association of the full-length SEL1L (Fig. 4E). In contrast, SEL1L^348–497^-KDEL and the single-residue mutation L521A in SEL1L^cent^ did not competitively inhibit the self-association of full-length SEL1L (Fig. 4E,F). These data suggest that the SEL1L forms self-oligomers and the oligomerization is mediated by the SEL1L^cent^ domain *in vivo*.

### Structural Comparison of SEL1L SLRs with TPRs or SLRs of Other Proteins

**Figure 5:**
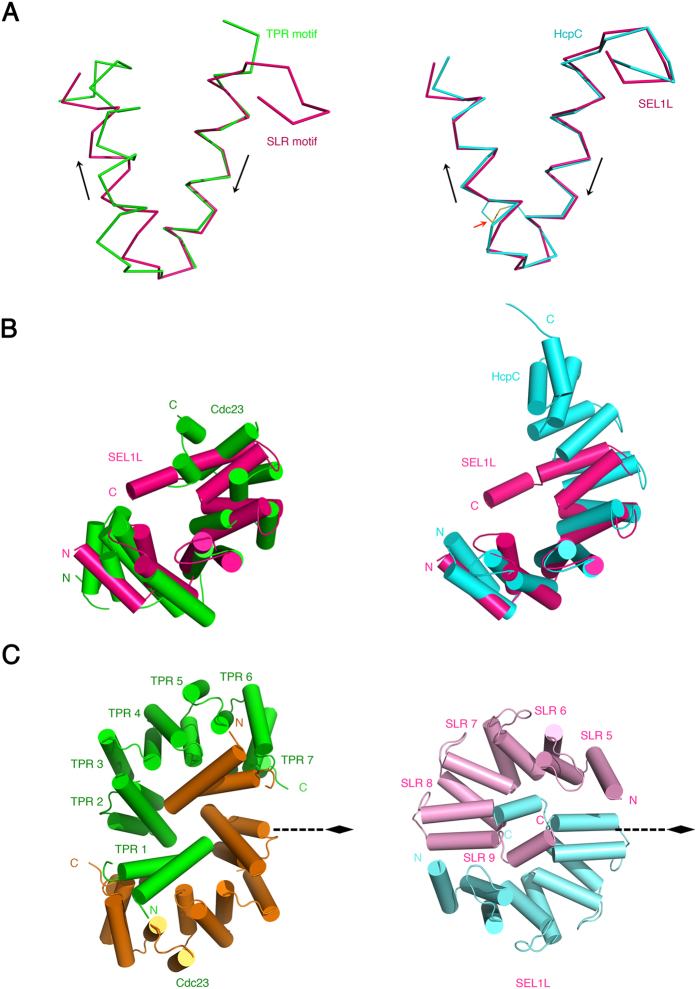
Comparison of SLR in SEL1L with TPR or Other SLR-Containing Proteins. (**A**) Ribbon diagram showing superimposition of an isolated TPR motif from Cdc23 and an SLR motif from SEL1L^cent^ (left), and SLR motifs in HcpC and SEL1L^cent^ (right). The SEL1L, Cdc23, and HcpC are colored magenta, green and cyan, respectively. Black arrows indicate the helical axes. The red arrow indicates disulfide bonds in the HcpC, and Cys residues involved in disulfide bonding are shown by a yellow line. (**B**) Ribbon representation showing superimposition of Cdc23 and SEL1L^cent^ (left) or HcpC and SEL1L^cent^ (right) to compare the overall organization of the α-solenoid domain. Both SEL1L^cent^ schematics are identically oriented for comparison. The Cα atoms of the residues in each α-solenoid domain are superimposed with a root-mean-squared deviation of 3.3 Å for Cdc23 and SEL1L^cent^ (left), and 2.5 Å for HcpC and SEL1L^cent^ (right). SEL1L^cent^, Cdc23, and HcpC are colored as in (**A**). (**C**) Ribbon diagram showing the overall structure of Cdc23^N-term^ (left) and SEL1L^cent^ (right) to compare their similarities regarding dimer formation through domain swapping. The view is along the two-fold axis.

Previous studies reveal that TPRs and SLRs have similar consensus sequences, suggesting that their three-dimensional structures are also similar^18^. The superposition of isolated TPRs from Cdc23 (*S. pombe*, cell division cycle 23 homolog, PDB code 3ZN3) and SLRs from HcpC (Helicobacter Cysteine-rich Protein C, PDB code 1OUV) yields RMSDs below 1 Å, confirming that the isolated repeats are indeed similar^20^. This is relevant to SLR motifs in SEL1L, as isolated SLR motifs from SEL1L^cent^ showed good structural alignment with isolated TPRs (RMSD 1.6 Å for all Cα chains) from Cdc23^N-term^ and SLRs (RMSD 0.6 Å for all Cα chains) from HcpC (Fig. 5A). However, superimposing the structure of SLR motifs 5 to 9 from SEL1L^cent^ onto the overall Cdc23^N-term^ or full-length HcpC structures revealed that SLR motifs 5 to 9 in SEL1L^cent^ have a different superhelical structure than either Cdc23 or HcpC (RMSD values of >2.5 Å for Cα atoms) (Fig. 5B). The differences may result from the differing numbers of residues in the loops and differences in antiparallel helix packing. Moreover, there are conserved disulfide bonds in the SLR motifs of HcpC and HcpB, but no such bonds are observed in SEL1L^cent^. These factors contribute to the differences in the overall conformation of the SLR motifs in SEL1L and other SLR or TPR motif-containing proteins.

Another major difference in the structure of SLR motifs between SEL1L and HcpC is the oligomeric state of proteins. The TPR motif is involved in the dimerization of proteins such as Cdc23, Cdc16, and Cdc27^26^. In particular, the N-terminal domain of Cdc23 (Cdc23^N-term^) has a TPR-motif organization similar to that of the SLR motif in SEL1L^cent^. The seven TPR motifs of Cdc23^N-term^ are assembled into a superhelical structure, generating a hollow surface and encircling its dimer counterpart in an interlocking clasp-like arrangement (Fig. 5C)^26^. The TPR motif 1 (TPR1) of each Cdc23^N-term^ subunit is located in the hollow surface of the counter subunit and interacts with residues lining the inner groove TPR α-helices, generating two-fold symmetry homotype interactions. However, in this structure, a conformational change in the TPR motif itself is not observed.

Self-association of HcpC has not been reported, and there is no domain-swapped structure in the SLR motifs of HcpC, in contrast to that observed in SEL1L^cent^. Although SEL1L contains a number of SLR motifs comparable to HcpC, the SLR motifs in SEL1L are interrupted by other sequences, making three SLR motif clusters (Fig. 1A). The interrupted SLR motifs may be required for dimerization of SEL1L^cent^, as five SLR motifs are more than enough to form the semicircle of the yin-yang symbol (Fig. 1B). Helix 5A from SLR motif 5 meets helix 9A from SLR motif 9 of the counterpart SEL1L. If the SLR motifs 5 to 9 were not isolated from other SLR motifs, steric hindrance could interfere with dimerization of SEL1L. This is one of the biggest differences from TPRs in Cdc23 and from the SLRs in HcpC, where the motifs exist in tandem. TPR and SLR motifs are generally involved in protein-protein interaction modules, and the sequences between the SLR motifs of SEL1L might actually facilitate the self-association of this protein.

### SLR-C of SEL1L Binds HRD1 N-terminus Luminal Loop

**Figure 6:**
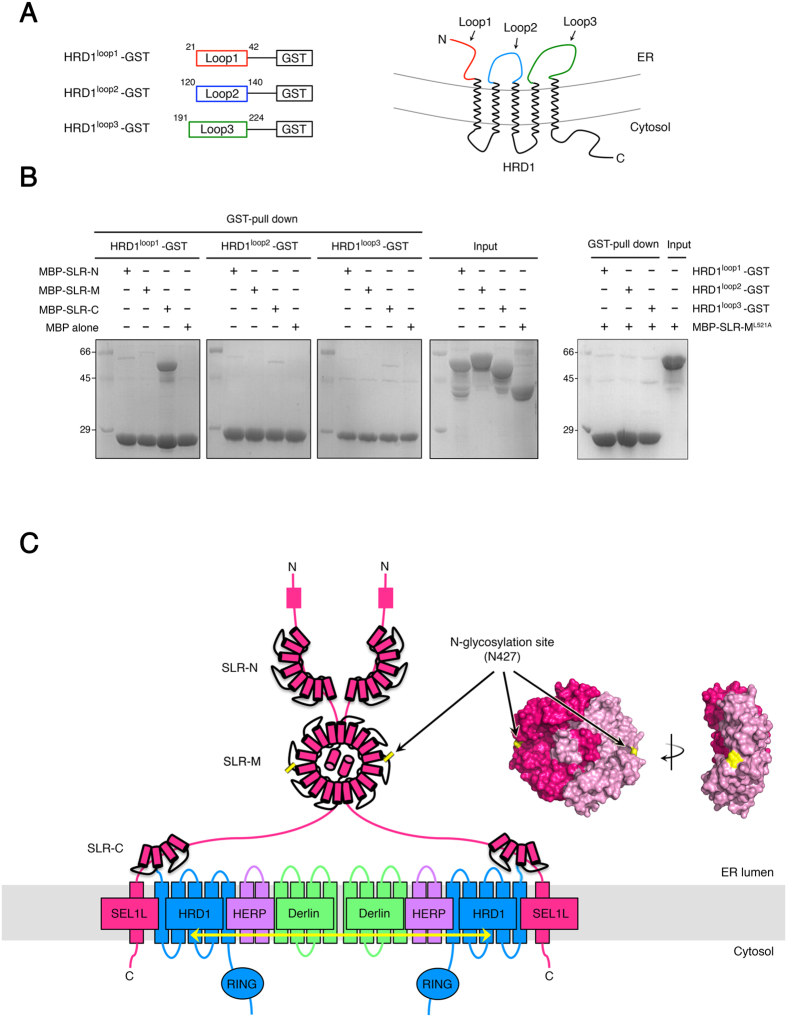
The Role of SLR-C in ERAD machinery and Model for the Organization of Proteins in Membrane-Associated ERAD Components. (**A**) Schematic diagram shows three HRD1 fragment constructs used in the GST pull-down experiment. (**B**) Pull-down experiments to examine the interactions between HRD luminal loops and certain SLR motifs of SEL1L. Fragments of the luminal loop of HRD1 fused to GST were immobilized on glutathione sepharose beads and incubated with purified three clusters of SLR motifs and monomer form of SLR-M (SLR-M^L521A^, right panel) in SEL1L. Proteins were analyzed by 12% SDS-PAGE and Coomassie blue staining. (**C**) Schematic representation of the organization of metazoan ERAD components in the ER membrane. The 11 SLR motifs of SEL1L were expressed with red cylinders and grouped into three parts (SLR-N, SLR-M, and SLR-C) based on the sequence alignment across the motifs and the crystal structure presented herein. We hypothesized that the interrupted SLR motifs of SEL1L have distinct functions such that the SLR-M is important for dimer formation of the protein, and SLR-C is involved in the interaction with HRD1 in the ER lumen. The surface representation of SEL1L^cent^ is placed in the same orientation as that shown in the schematic model to show that the putative N-glycosylation site, residue N427 (indicated in yellow), is exposed on the surface of the protein. The yellow arrow indicates self-association among the respective components.

Based on the structural data presented herein, a possible arrangement of membrane-associated ERAD components in mammals, highlighting the molecular functions of SLR domains in SEL1L, is shown in Fig. 6C. We suggest that the middle SLR domains are involved in the dimerization of SEL1L based on the crystal structure and biochemical data. SLR-C, which contains SLR motifs 10 to 11, might be involved in the interaction with HRD1. Indirect evidence from a previous yeast study shows that the circumscribed region of C-terminal Hrd3p, specifically residues 664–695, forms contacts with the Hrd1 luminal loops^12^. The Hrd3p residues 664–695 correspond to mouse SEL1L residues 696–727, which include the entire helix 11B (residue 697–709) of SLR motif 11 and a well-conserved adjacent region (Supplementary Fig. 4). This observation is supported by the following: (1) the meticulous range of SLR motif 10 to 11 is newly established from a structure-guided SLR motif alignment, based on the present structure study, and (2) the relatively high sequence conservation between mammalian SEL1L and yeast Hrd3p around SLR motifs 10 to 11, which contain contact regions with HRD1 (Hrd1p) (Supplementary Figs. 4 and 5). To address this hypothesis, we prepared constructs encoding mouse HRD1 luminal fragments fused to GST as shown in Fig. 6A, and tested their ability to bind certain SLR motifs in SEL1L. The fusion proteins were immobilized on glutathione-Sepharose beads and probed for binding to SLR-N, SLR-M, SLR-C, and monomer form of SLR-M (SLR-M^L521A^). Figure 6B shows that the SLR-C, consisting of SLR motifs 10 and 11, exclusively interacts with N-terminal luminal loop (residues 21–42) of HRD1.

The molecular functions of SLR-N are unclear. One possibility is that SLR-N contributes to substrate recognition of proteins to be degraded because there are a couple of putative glycosylation sites within the SLR-N domain (Fig. 1A). SEL1L^cent^ contains a putative N-glycosylation site, Asn 427, which is highly conserved among different species and structurally exposed to the surface of the SEL1L dimer according to the crystal structure (Fig. 6C).

## Discussion

Many reports demonstrate that membrane-bound ERAD machinery proteins in yeast, such as Hrd1p, Der1p, and Usa1p, are involved in oligomerization of ERAD components^27^,^28^,^29^. The Hrd1p complex forms dimers upon sucrose gradient sedimentation^5^,^30^ and size-exclusion chromatography^30^. Previous data show that HA-epitope-tagged Hrd3p or Hrd1p efficiently co-precipitate with unmodified Hrd3p and Hrd1p, respectively, suggesting that both Hrd1p and Hrd3p homodimers are involved in self-association of the Hrd complex. Considering that the functional and structural composition of ERAD components are conserved in both yeast and mammals, we propose that the mammalian ERAD components also form self-associating oligomers. This hypothesis is supported by cross-linking data suggesting that human HRD1 forms a homodimer^31^. Consistent with the previous data, our crystal structure and biochemical data demonstrate that mouse SEL1L^cent^ exists as a homodimer in the ER lumen via domain swapping of SLR motif 9. We need to further test whether there are contacts involved in dimer formation in SEL1L in addition to those in the SLR-M region.

In yeast, Usa1p acts as a scaffold for Hrd1p and Der1p, in which the N-terminus of Usa1p interacts with the C-terminal 34 amino acids of Hrd1p in the cytosol to induce oligomerization of Hrd1p, which is essential for its activity^30^,^32^. However, metazoans lack a clear Usa1p homolog. Although mammalian HERP has sequences and domains that are conserved in Usa1p, the molecular function of HERP is not clearly related to that of Usa1p^4^,^31^. Rather, recent research shows that a transiently expressed HRD1-SEL1L complex alone associates with the ERAD lectins OS9 or XTP-B and is sufficient to facilitate the retrotranslocation and degradation of the model ERAD substrate α-antitrypsin null Hong-Kong (NHK) and its variant, NHK-QQQ, which lacks the N-glycosylation sites^33^. Assuming that the correct oligomerization of ERAD components may be critical for their function, we hypothesize that homodimer formation of SEL1L in the ER lumen may stabilize oligomerization of the HRD complex, given that SEL1L forms a stoichiometric complex with HRD1^10^,^13^,^15^,^30^. This is further supported by our data showing that the SLR-C of SEL1L directly interacts with the luminal fragment of HRD1 in the ER lumen.

Although the organization of membrane-bound HRD complex components may be very similar between metazoans and yeast, the molecular details of interactions between the components may not necessarily be conserved. In yeast, it is unclear whether self-association of Hrd3p is due to SLR motifs because the sequence of Hrd3p does not align precisely with the SLR motifs in SEL1L^18^. Furthermore, we are uncertain whether self-association of Hrd3p contributes to formation of the active form of the Hrd1p complex. Recently, a truncated version of Yos9 was shown to form a dimer in the ER lumen and to contribute to the dimeric state of the Hrd1p complex^34^. This interaction seems to be weak because direct Yos9-Yos9 interactions were not detected in immunoprecipitation experiments from yeast cell extracts containing different epitope-tagged variants of Yos9. However, the dimerization of Yos9 could provide a higher stability for the Hrd1p complex oligomer. Likewise, the dimerization of SEL1L might provide stability for the mammalian HRD oligomer complex. Further cell biological studies are required to clarify whether SEL1L (Hrd3p) dimerization could be cooperative with the oligomerization of the HRD complex.

Considering that it is very important for the function of the HRD complex that the components assemble as oligomers, we believe that the self-association of SEL1L strongly contributes to generating active forms of the HRD complex, even in the absence of Usa1p, in metazoans. These findings should provide a foundation for molecular-level studies to understand the membrane-associated HRD complex assembly in ERAD.

## Methods

### Protein Production

The expression and purification of SEL1L was performed as described previously^35^.

### Crystallization and SAD Structure Determination of SEL1L^cent^

Crystals were grown using the hanging-drop vapor diffusion method at 4 °C. For crystallization of the *M. musculus* SEL1L^cent^, 1 μl of protein solution (in 25 mM Tris-HCl, 150 mM NaCl, and 5 mM DTT, pH 7.5) was equilibrated with 1 μl of well solution (30% isopropanol, 100 mM NaCl, 100 mM Tris, 5 mM DTT, and 20 mM phenol, pH 8.5). The crystals, which appeared after 4 days, contain two SEL1L^cent^ dimers in the asymmetric unit (space group P2_1_, a = 29.13, b = 110.52, c = 109.81 Å, α = 90.00, β = 90.61, γ = 90.00, 44% solvent). For X-ray diffraction experiments, crystals were transferred to well solution plus paraffin-oil, then flash frozen in liquid nitrogen.

SAD data were collected with a Se-Met crystal at beamline 7A of the Pohang Accelerator Laboratory (PAL) and processed using HKL2000 software^36^. Native data (2.6 Å resolution) were collected from a single frozen crystal at the same beamline of PAL and were integrated and scaled as described above. The SAD data analysis was performed using Phenix software^37^ using data between 50 and 2.9 Å resolution. Phenix identified 31 of the 32 selenium sites and refined these to give a mean f.o.m. = 0.472. Electron density modification, including non-crystallographic symmetry (NCS) averaging, using the RESOLVE software^38^ yielded an initial electron density map of excellent quality. Model building and refinement were carried out with the Coot^39^ and Phenix programs, respectively. The final model was refined to an R factor of 20.7% (R_free_ = 27.7%) for native data between 30 and 2.6 Å resolution (Table 1). The final model consisted of 5402 protein atoms and 47 water molecules. There were no outliers in a Ramachandran plot of the final model. The model contained four copies of SEL1L^cent^ (residues 348–533) in the asymmetric unit. Of these, the following residues were not modeled due to weak electron densities: SEL1L^cent^ residues 348–351, 420, 421, and 525–533 in the first copy; residues 348–351 and 525–533 in the second and third copies; and residues 348–352 and 525–533 in the fourth copy. The X-ray data and refinement statistics are summarized in Table 1.

### Cell Culture and Plasmids Construction

HEK293T cells were cultured in DMEM (Gibco) supplemented with 10% FBS. The mouse *Sel1L* gene was cloned into pCS108 and the 3 × HA or 3 × FLAG tag was fused to the C-terminus of SEL1L. The signal peptide from *Xenopus* Sel1L was cloned into pCS108 and the mouse SEL1L^cent^ domain, SEL1L (348-497) fragments, and SEL1L^cent^ (L521A) were fused to the C-terminus of the signal peptide. Then, a 3 × HA or a 3 × FLAG tag was fused to the C-terminus of the constructs. For the ER retention signal, the KDEL sequences were added to the C-terminus of the fragments. The plasmids were transfected using Lipofectamine 2000 (Life Technologies) according to the manufacturer’s manual.

### Western Blot Analysis and Immunostaining

For western blot analysis, HEK293T cells were transfected with the indicated construct and harvested after washing in PBS. The cells were homogenized in lysis buffer (50 mM Tris, pH 7.4, 150 mM NaCl, 0.1% Triton X-100, 5% glycerol), supplemented with protease and phosphatase inhibitor cocktails. Homogenates were cleared by centrifugation at 13,200 rpm for 15 minutes at 4 °C. The lysates were subsequently used for either co-immunoprecipitation experiment or western blot analysis. For the western blot analysis, the samples were run onto 6–12% polyacrylamide gel. Blots were blocked in 5% TBS + 0.05% Tween 20 and incubated with anti-DDDD-K (Abcam) or anti-HA (Roche) antibodies. Proteins were visualized using HRP-conjugated secondary antibodies (1:4000) and SuperSignal West Pico Chemiluminescent Substrate or SuperSignal West Dura Extended Duration Substrate (Thermo) and exposed to ChemiDoc MP (Bio-Rad).

For immunostaining, the cells were fixed in 4% formaldehyde and incubated with the indicated antibodies. The coverslips were incubated in blocking solution (10% FBS + 2% DMSO in TBS + 0.1% Triton X-100) at room temperature for 30 minutes to block non-specific binding. Fluorescent labeling was performed using Alexa Fluor 555 or 488-conjugated secondary antibodies and nuclei were stained with DAPI. The samples were mounted and confocal images were obtained using a Zeiss LSM700.

### GST Pull-down Assay

For pull-down experiments, 400 μg of HRD1 luminal fragment GST-fusion proteins were incubated with 5 μl of a 50% (v/v) slurry of glutathione sepharose 4B beads (GE Healthcare) for 50 min at 4 °C. Beads were washed twice with buffer A (150 mM NaCl, 25 mM sodium phosphate pH 7.5, 5 mM DTT), and then mixed with 100 μg of MBP-SEL1L protein (SLR-N, SLR-M, SLR-C, and SLR-M^L521A^) in buffer A, in a total assay volume of 500 μl. The assay mix was incubated at 4 °C for 15 minutes, and beads were washed twice with 500 μl buffer A. Proteins were eluted with SDS sample buffer, and analyzed by SDS-PAGE.

## Additional Information

**Accession Numbers:** The coordinates and structure factors have been deposited in the Protein Data Bank with the accession code of 5B26.

**How to cite this article**: Jeong, H. *et al.* Crystal structure of SEL1L: Insight into the roles of SLR motifs in ERAD pathway. *Sci. Rep.* **6**, 20261; doi: 10.1038/srep20261 (2016).

## Supplementary Material

Supplementary Information

## Figures and Tables

**Table 1:** Data Collection and Refinement Statistics.

	SEL1L^cent^	
Data set:	Native	Se-SAD
PDB accession #:	5B26	
X-ray source	Beamline 7A, PAL	Beamline 7A, PAL
Temperature (K)	100	100
Space group:	P2_1_	P2_1_
Cell parameters a, b, c (Å)	29.13, 110.52, 109.81	29.51, 110.49, 109.81
	90.00, 90.61, 90.00	90.00, 90.74, 90.00
**Data processing**		
Wavelength (Å)	1.00000	0.97923
Resolution (Å)	50-2.60	50–2.90
R_merge_ (%)^a^	6.1 (38.7)*	9.4 (40.6)
I/σ	29.4 (4.6)	21.0 (3.3)
Completeness (%)	99.5 (99.3)	99.9 (100.0)
Redundancy	4.1 (4.1)	3.8 (3.8)
Measured reflections	88070	116951
Unique reflections	21479	30823
**Refinement statistics**		
Data range (Å)	30-2.60	
Reflections	21446	
Nonhydrogen atoms	5402	
Water molecules	47	
R.m.s. ∆ bonds (Å)^b^	0.010	
R.m.s. ∆ angles (°)^b^	1.365	
R-factor (%)^c^	20.7	
R_free_ (%)^c^^,d^	27.7	
Ramachandran plot, residues in		
Most favored regions (%)	92.8	
Additional allowed regions (%)	6.5	
Generously allowed regions (%)	0.7	
Disallowed regions (%)	0.0	

*Highest resolution shell is shown in parenthesis.

^a^R_merge_ = 100 × ∑_h_∑_i_ | *I*_i_(h) − <*I*(h) >|/∑_h_ <*I*(h)>, where *I*_i_(h) is the *i*th measurement and <*I*(h)> is the weighted mean of all measurement of *I*(h) for Miller indices h.

^b^Root-mean-squared deviation (r.m.s. ∆) from target geometries.

^c^R-factor = 100 × ∑|F_P_ – F_P(calc)_|/∑ F_P_.

^d^R_free_ was calculated with 5% of the data.
