# Supplementary material for: Crystal structure of SEL1L: Insight into the roles of SLR motifs in ERAD pathway
Source: Sci Rep. 2016 Feb 9;6:20261. doi: 10.1038/srep20261 (PMC4746701; doi:10.1038/srep20261)
Supplement: Supplementary Information [file srep20261-s1.pdf]

Supplementary file (Jeong et al.)

Crystal structure of SEL1L: Insight into the roles of SLR motifs in ERAD pathway

Hanbin Jeong[1], Hyo Jung Sim[1], Eun Kyung Song[1], Hakbong Lee[1], Sung-Chul Ha[2], Youngsoo Jun[3], Tae Joo Park[1*], Changwook Lee[1*]

[1]Department of Biological Sciences, School of Life Sciences, Ulsan National Institute of Science and Technology, 50 UNIST-gil, Ulsan 44919, Republic of Korea

[2]Pohang Accelerator Laboratory, Pohang University of Science and Technology, Pohang, Kyungbuk 37673, Korea

[3]School of Life Sciences, Gwangju Institute of Science and Technology, Gwangju 61005, Korea

[*]Correspondence regarding this manuscript to:

Tae Joo Park
Telephone: 82-52-217-2582
Fax: 82-52-217-2639
E-mail: parktj@unist.ac.kr

Changwook Lee
Telephone: 82-52-217-2534
Fax: 82-52-217-2639
E-mail: changwook@unist.ac.kr

Supplementary Information

Additional Contacts between SEL1L^{cent} Molecules in the Crystal Lattice

Supplementary Fig. 1 shows the overall organization of SEL1L^{cent} shown in the crystallographic unit cell to highlight additional contact sites between SEL1L^{cent} molecules. Four dimers of SEL1L^{cent} ([AB, CD] and the symmetry-related [A′B′ and C′D′]) are located in the same plane with overall dimensions of 150 × 150 × 35 Å, and are packed by protein-protein interactions. No significant differences between the two dimers (AB and CD) in the asymmetric unit were observed, with a final root mean square deviation (RMSD) of 0.8 Å for all Cα atoms.

PISA analysis suggested that there are three contact regions among four dimers of SEL1L^{cent} (Supplementary Fig. 1A) in the unit cells[1]. Helix 5A from SLR motif 5 and the additional loop from SLR motif 8 are mainly involved in crystal contacts I and II (Supplementary Fig. 1B). However, the interactions shown in contacts I and II are not conserved; rather, they are displaced from each other. In contact III, the Gly 456 and Gly 458 residues in helix 7B form hydrogen bonds with the same residues from a quasi centro-symmetry-related molecule (A′ and D′). Since the interactions observed in contact I, II and III are not conserved, and there is no regular relationship among dimers, we conclude that these interactions might not be due to biological contacts but instead due to crystal contacts. This observation agrees with the biochemical analytical ultracentrifugation and size-exclusion chromatography data for native and mutant SEL1L^{cent}, which show that the dimer form of SEL1L^{cent} is formed via contacts between SLR motif 9 within each monomer.

Supplementary Figures

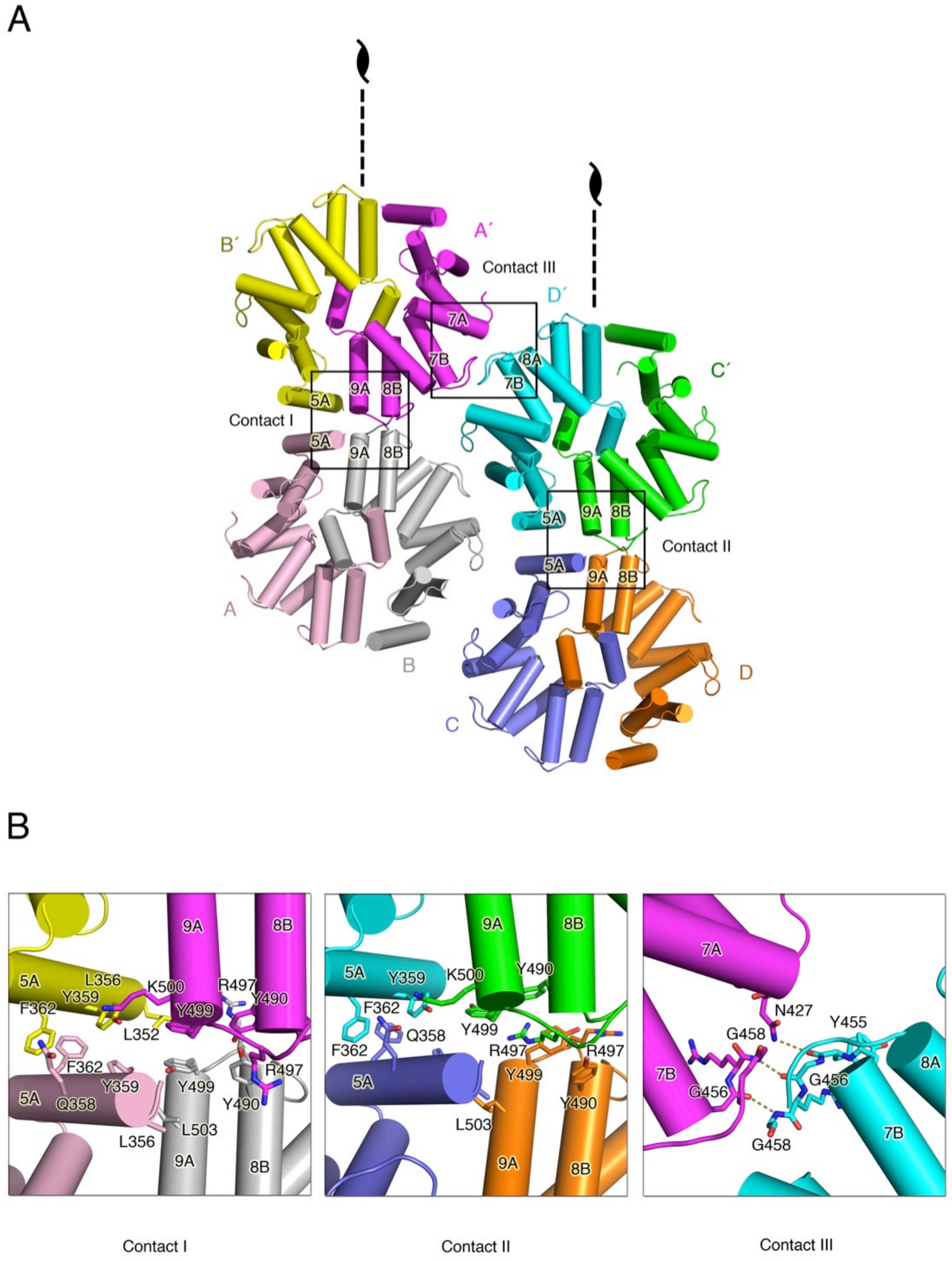

Supplementary Fig. 1. Protein organization in the Crystal Lattice

(A) Ribbon representation of SEL1L^{cent} in the crystal unit cell showing additional contacts between the four dimers. The three contact surfaces are marked with black boxes and labels. The α helices are represented by cylinders. The two-fold screw axis is indicated by the black dotted line and symbol.

(B) Close-up view showing the three contact surfaces indicated by the black boxes in (A). The residues involved in the interactions are depicted in stick model form. Oxygen atoms are shown in red, nitrogen in blue, and hydrogen bonds are indicated by yellow dotted lines.

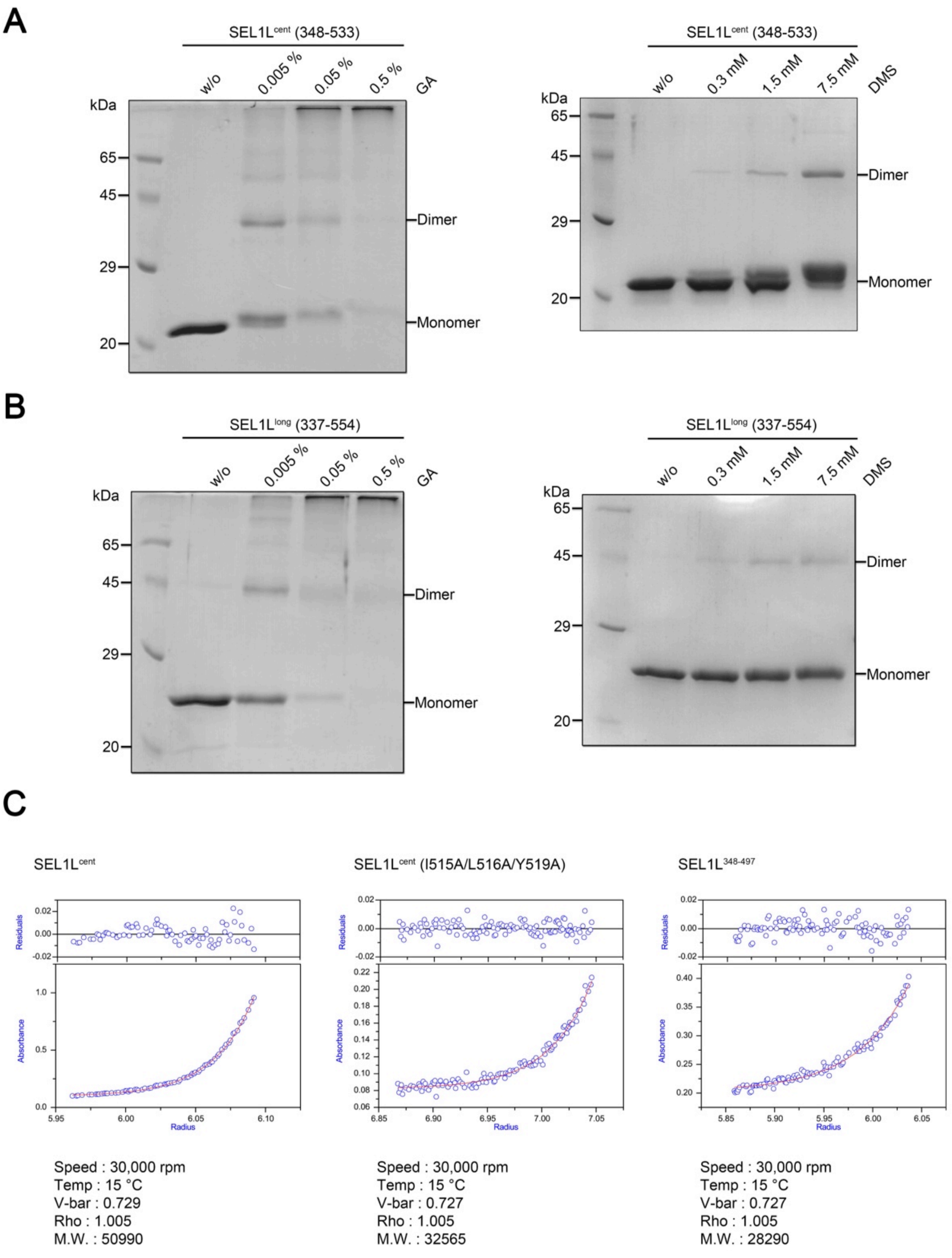

Supplementary Fig. 2. SEL1L Self-association Experiments

(A) Cross-linking experiment to show self-association of SEL1L residues 348-533. The 20 μM of proteins were cross-linked with glutaraldehyde (GA, left) or dimethyl suberimidate (DMS, right) at different concentration ranges.

(B) Cross-linking experiment to show self-association of SEL1L residues 337-554. The experiments were carried out as (A).

(C) Equilibrium fit results of the analytical ultracentrifuge for the wild type (left), triple point mutant (I515A, L516A, Y519A, middle), and deletion mutant (residues 348-497, right) SEL1L^{cent}. The lower panel depicts the fitted overlay (red line) to the experimental data (blue circles). The upper panel depicts the residuals.

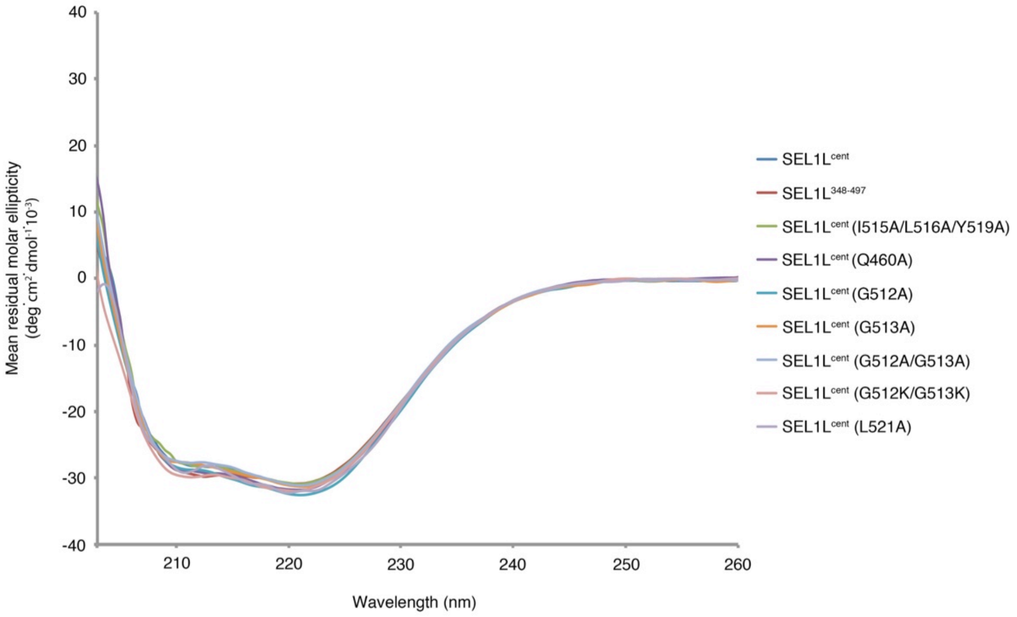

Supplementary Fig. 3. Circular Dichroism Spectroscopy

Analysis of conformation differences between the wild type and $SEL1L^{cent}$ mutants by circular dichroism (CD). CD spectroscopy measurement shows that all mutant proteins displayed identical patterns of spectra to that of wild type, indicating that no significant changes occurred in the structure of $SEL1L^{cent}$ upon mutation.

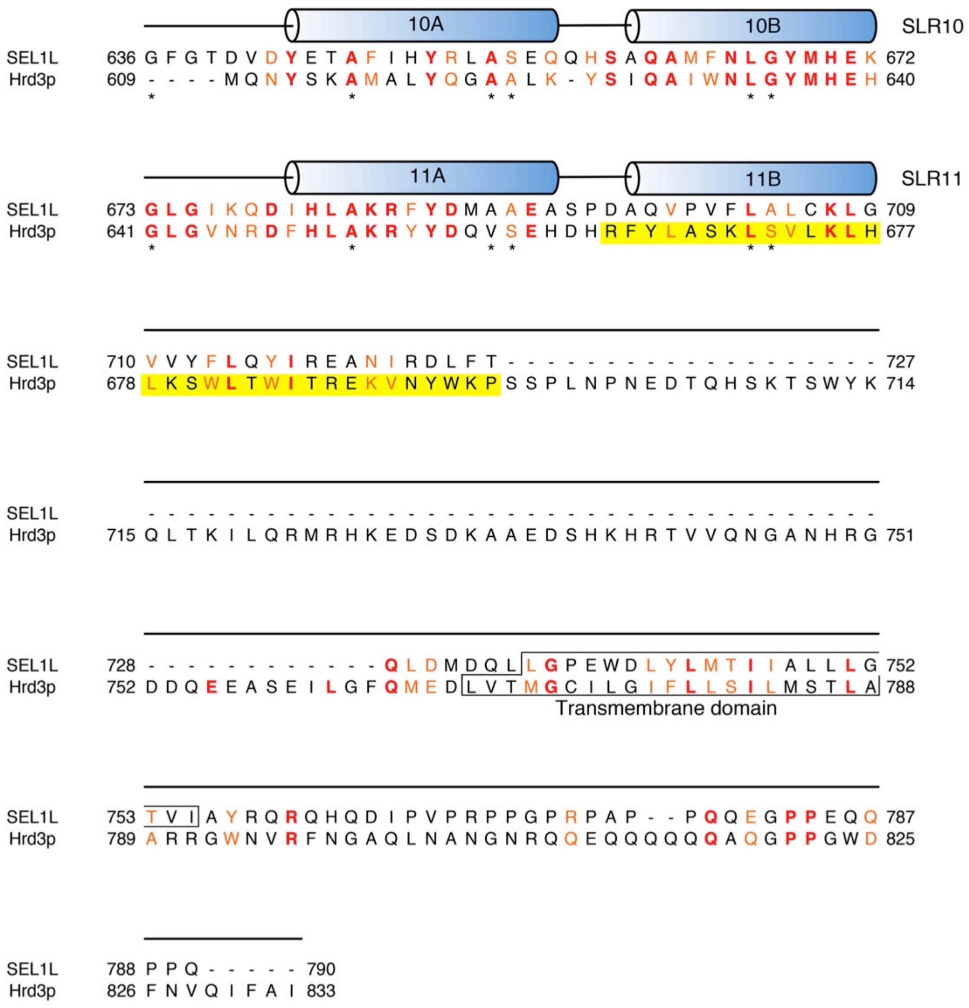

Supplementary Fig. 4. Sequence Comparison of the C-terminus of Mouse SEL1L and Yeast Hrd3p

The sequence of SEL1L (residues 636–790) was aligned with that of yeast Hrd3p (residues 609–833) to identify sequence similarities using CLUSTAL W[2]. Conserved or similar amino acids are shown in red or orange, respectively. The Hrd3p sequences involved in the interaction with Hrd1p are highlighted by the yellow box[3]. The secondary structure elements are indicated above the sequences, with helices depicted as cylinders. The asterisks below the sequence indicate the amino acids present in the regular SLR motif. Putative transmembrane domains in SEL1L and Hrd3p are indicated by the black square.

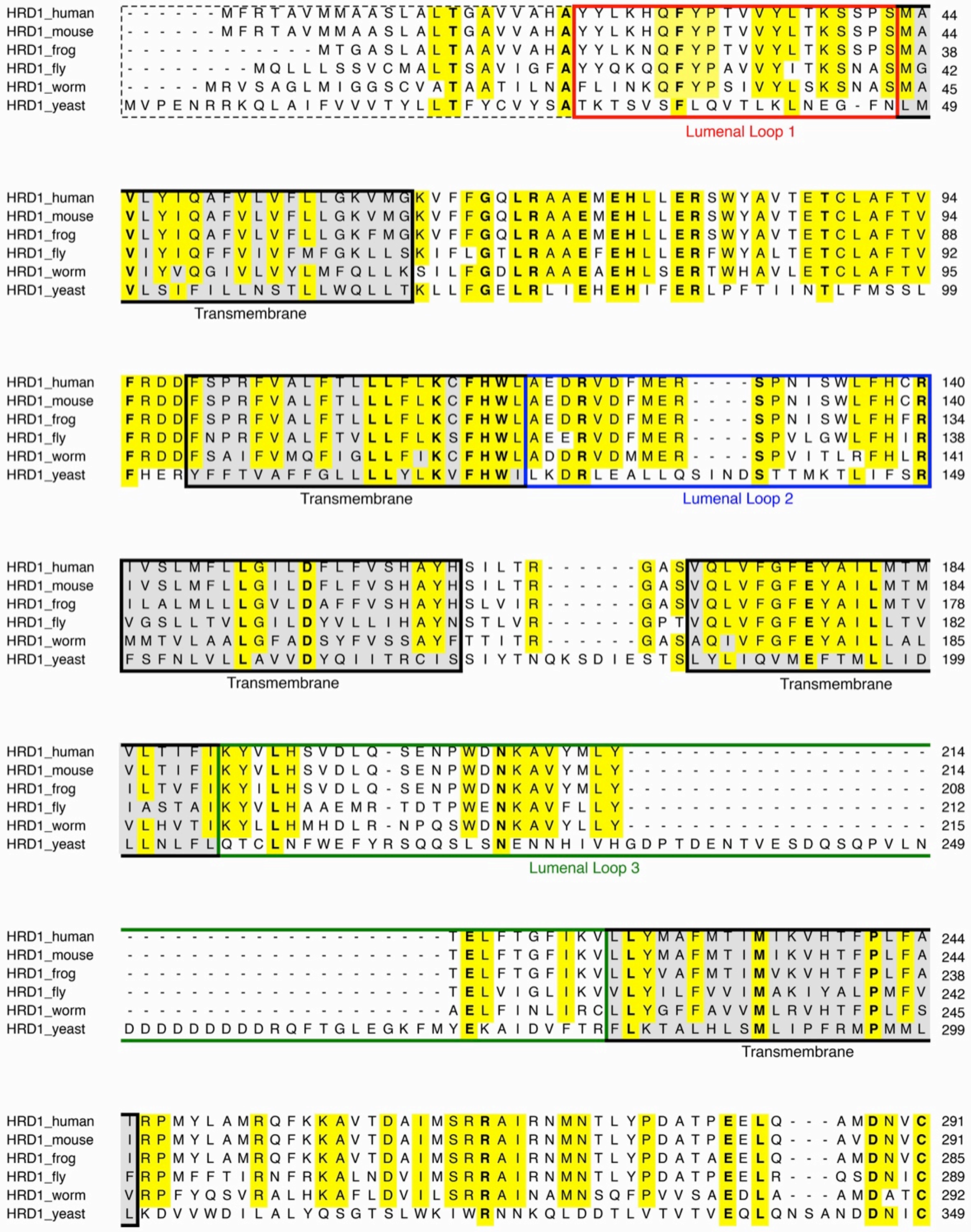

Supplementary Fig. 5. Sequence alignment of the transmembrane domain of HRD1

Sequence alignment of HRD1 from Homo sapiens (Q86TM6), Mus musculus (Q9DBY1), Xenopus laevis (Q6NRL6), Drosophilar melanoganster (Q95SP2), Caenorhabditis elegans (Q20798), and Saccharomyces cerevisiae (Q08109). Residues that are conserved in at least five of the six sequences are shaded yellow. Color boxes indicate three luminal fragments used in the GST pull-down experiments.

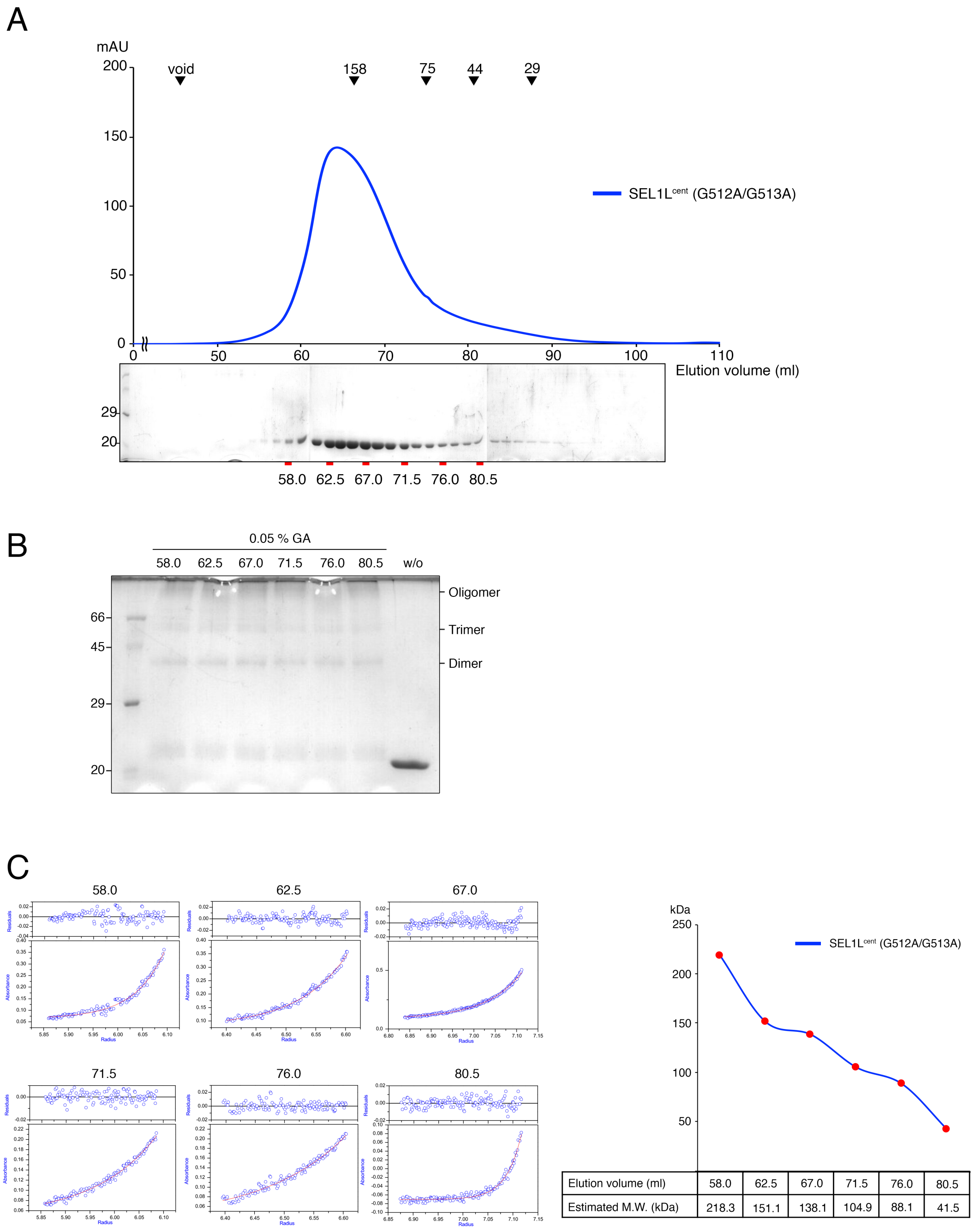

Supplementary Fig. 6. G512A/G513A mutation of SEL1L^{cent} induces the proteins to polydisperse in solution

(A) Size-exclusion chromatography profile and the corresponding SDS-PAGE were shown.

Size-exclusion chromatography was conducted as described in Fig. 2B. SEL1L (G512A/G513A) eluted over a broad range from the gel-filtration column. The standard molecular masses are shown at the top as in Fig. 2B. Red square indicates the fractions used in biochemical experiments below.

(B) Cross-linking experiment to show self-association of SEL1L^{cent} (G512A/G513A) at different elution volumes (58, 62.5, 67, 71.5, 76, 80.5 ml) from (A). The 20 μM of proteins were cross-linked with 0.05 % glutaraldehyde, and separated by SDS-PAGE. No significant differences were shown among cross-linked products from proteins at different elution volumes.

(C) Equilibrium fit results of the analytical ultracentrifuge for the each fraction at 58, 62.5, 67, 71.5, 76, 80.5 ml from the size-exclusion chromatography above. The right panel represents the estimated molecular weights of each fraction, based on analytical ultracentrifugation. The molecular weights are distributed between 218 to 41.5 kDa. The data shows that the molecular weights of SEL1L^{cent} (G512A/G513A) were not correlated to the elution volumes, but instead showed an irregular distribution, voiding it from any meaningful self-association.

Supplementary Materials and Methods

Chemical Cross-linking Analysis

20 μM of mouse SEL1L proteins (SEL1L^{cent}, residues 348-533 and SEL1L^{long}, residues 337-554) were prepared in 25 mM Hepes (pH 7.5), 150 mM NaCl, 5 mM DTT. The proteins were incubated with glutaraldehyde (GA, sigma) or dimethyl suberimidate (DMS, sigma) across the various concentration ranges (0.005%, 0.05% and 0.5% for GA, 0.3 mM, 1.5 mM and 7.5 mM for DMS) for 30 min at 18°C. The reactions were terminated with 100 mM Tris and the products were subjected to SDS-PAGE.

Analytical Ultracentrifugation

The molecular mass of the native and mutant proteins of SEL1L^{cent} was analyzed by analytical ultracentrifugation (Optima XL-A; Beckman) using the sedimentation equilibrium technique. For sedimentation equilibrium analytical ultracentrifugation, the protein samples were prepared in buffer containing 25 mM Tris-HCl (pH 7.5), 200 mM NaCl, and 5 mM β-mercaptoethanol with the concentrations of 10 μM, 15 μM, and 20 μM. Data were evaluated using a nonlinear least-squares curve-fitting algorithm (XL-A data analysis software). For the equilibrium analysis, scans at equilibrium from multiple speeds (10000, 20000 and 30000 rpm) were collected at 15°C using an An60Ti rotor (Beckman) and by measuring absorbance at 280 nm. The measurements were fit to a single species model using the Origin 6.03 software (Beckman Coulter, Inc.).

Circular Dichroism Analysis

Structural changes in the SEL1L^{cent} mutants (5 μM) versus the wild type SEL1L^{cent} proteins were monitored by a circular dichroism (CD) spectrometer (Jasco J-815) at various

wavelengths (200-260 nm). All the samples used here were prepared in 25 mM Tris, 150 mM NaCl, 5 mM β-mercaptoethanol, pH 7.5.

Supplementary References

1 Krissinel, E. & Henrick, K. Inference of macromolecular assemblies from crystalline state. *J Mol Biol* **372**, 774-797 (2007).

2 Thompson, J. D., Higgins, D. G. & Gibson, T. J. CLUSTAL W: improving the sensitivity of progressive multiple sequence alignment through sequence weighting, position-specific gap penalties and weight matrix choice. *Nucleic Acids Res* **22**, 4673-4680 (1994).

3 Gauss, R., Jarosch, E., Sommer, T. & Hirsch, C. A complex of Yos9p and the HRD ligase integrates endoplasmic reticulum quality control into the degradation machinery. *Nat Cell biol* **8**, 849-854 (2006).
